# Supplementary material for: Lipidomic analysis of bile from patients with extrahepatic cholangiocarcinoma
Source: PLoS One. 2026 Mar 19;21(3):e0345136. doi: 10.1371/journal.pone.0345136 (PMC13001927; doi:10.1371/journal.pone.0345136)
Supplement: S1 Table — (DOCX) [file pone.0345136.s001.docx]

**S1 Table. Individual Clinicopathological Characteristics of Patients with Extrahepatic Cholangiocarcinoma (n = 12)**

| Case | Tumor Size (cm) | Location | T Stage | N Stage | M Stage | AJCC  Stage | Histology | Treatment |
| --- | --- | --- | --- | --- | --- | --- | --- | --- |
| 1 | 2.5 | distal CBD | 3 | 0 | 0 | 4 | Adenocarcinoma | CTx |
| 2 | 1.8 | Hilar, type II | 2 | 0 | 1 | 4 | Adenocarcinoma | CTx |
| 3 | 2.4 | distal CBD | 2 | 1 | 1 | 4 | Adenocarcinoma | CTx |
| 4 | 1.6 | distal CBD | 3 | 1 | 0 | 3 | Adenocarcinoma | CTx |
| 5 | 2.2 | distal CBD | 2 | 2 | 0 | 4 | Adenocarcinoma | CTx |
| 6 | 0.6 | distal CBD | 1 | 0 | 0 | 1 | Adenocarcinoma | Surgery |
| 7 | 2.6 | Hilar, type IV | 4 | 0 | 0 | 4 | Adenocarcinoma | CTx |
| 8 | 3.4 | CHD | 3 | 0 | 1 | 4 | Adenocarcinoma | CTx |
| 9 | 1.5 | CHD | 1 | 0 | 0 | 1 | Adenocarcinoma | Surgery |
| 10 | 2.7 | Hilar, type IV | 4 | 0 | 0 | 4 | Adenocarcinoma | CTx |
| 11 | 5.9 | distal CBD | 3 | 1 | 0 | 3 | Adenocarcinoma | Surgery |
| 12 | 2.2 | distal CBD | 2 | 1 | 0 | 3 | Adenocarcinoma | Surgery |

Abbreviations: CHD, common hepatic duct; CBD, common bile duct; CTx, chemotherapy
