# Supplementary figures and images for: Lipidomic analysis of bile from patients with extrahepatic cholangiocarcinoma
Source: PLoS One. 2026 Mar 19;21(3):e0345136. doi: 10.1371/journal.pone.0345136 (PMC13001927; doi:10.1371/journal.pone.0345136)

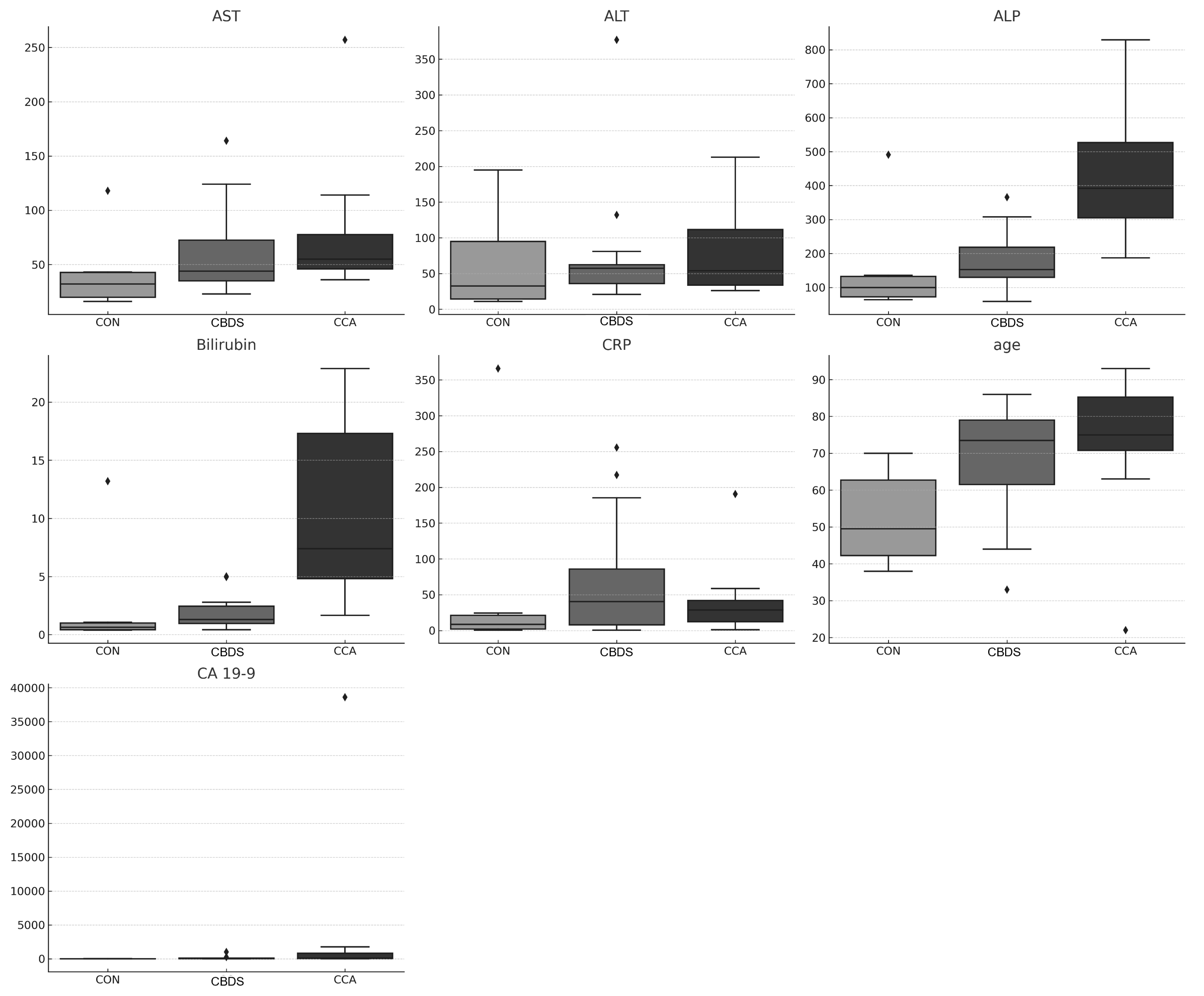

Supplement: S1 Fig — (TIF) [file pone.0345136.s002.tif]

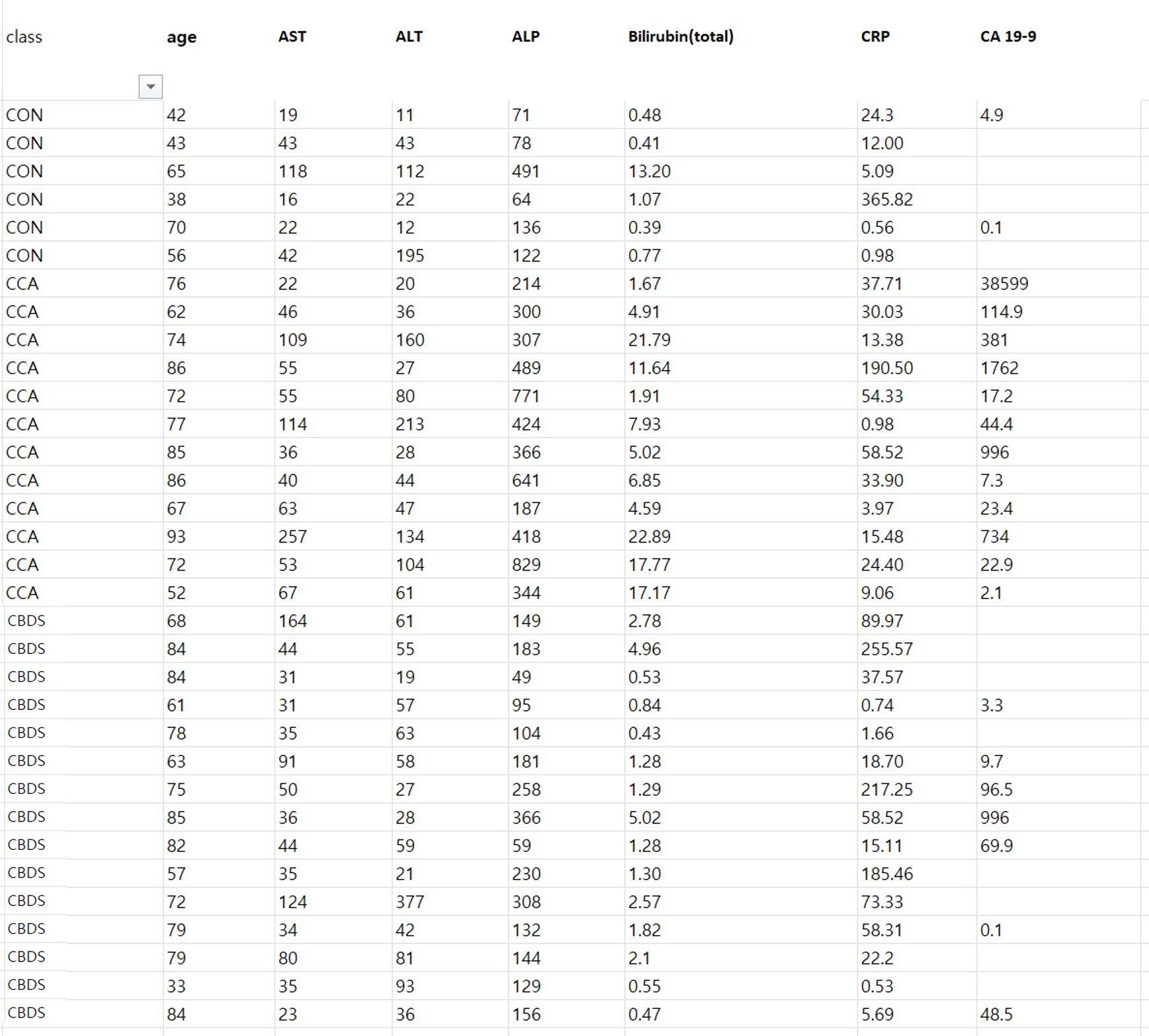

Supplement: S2 Fig — (TIF) [file pone.0345136.s003.tif]

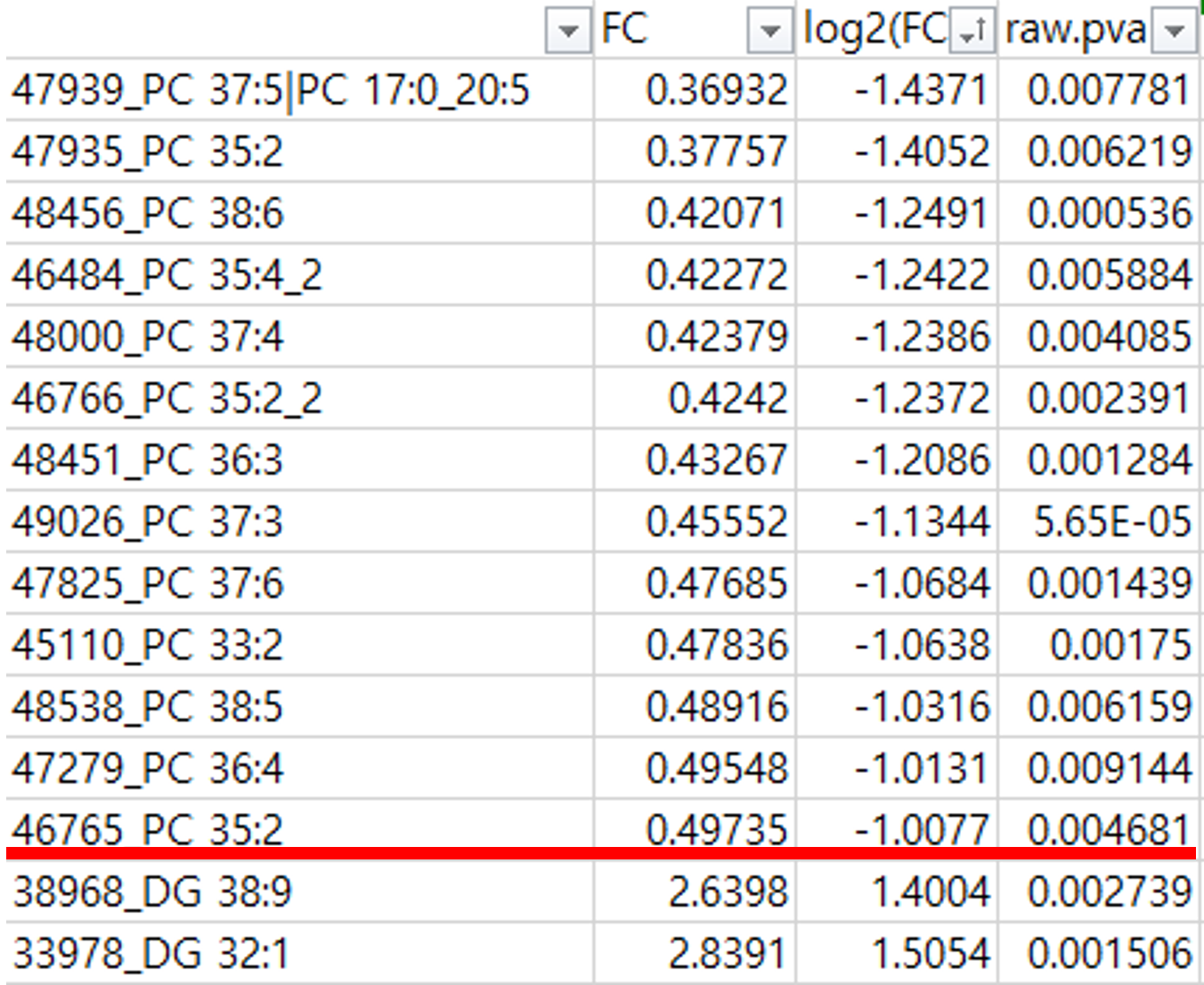

Supplement: S3 Fig — (TIF) [file pone.0345136.s004.tif]

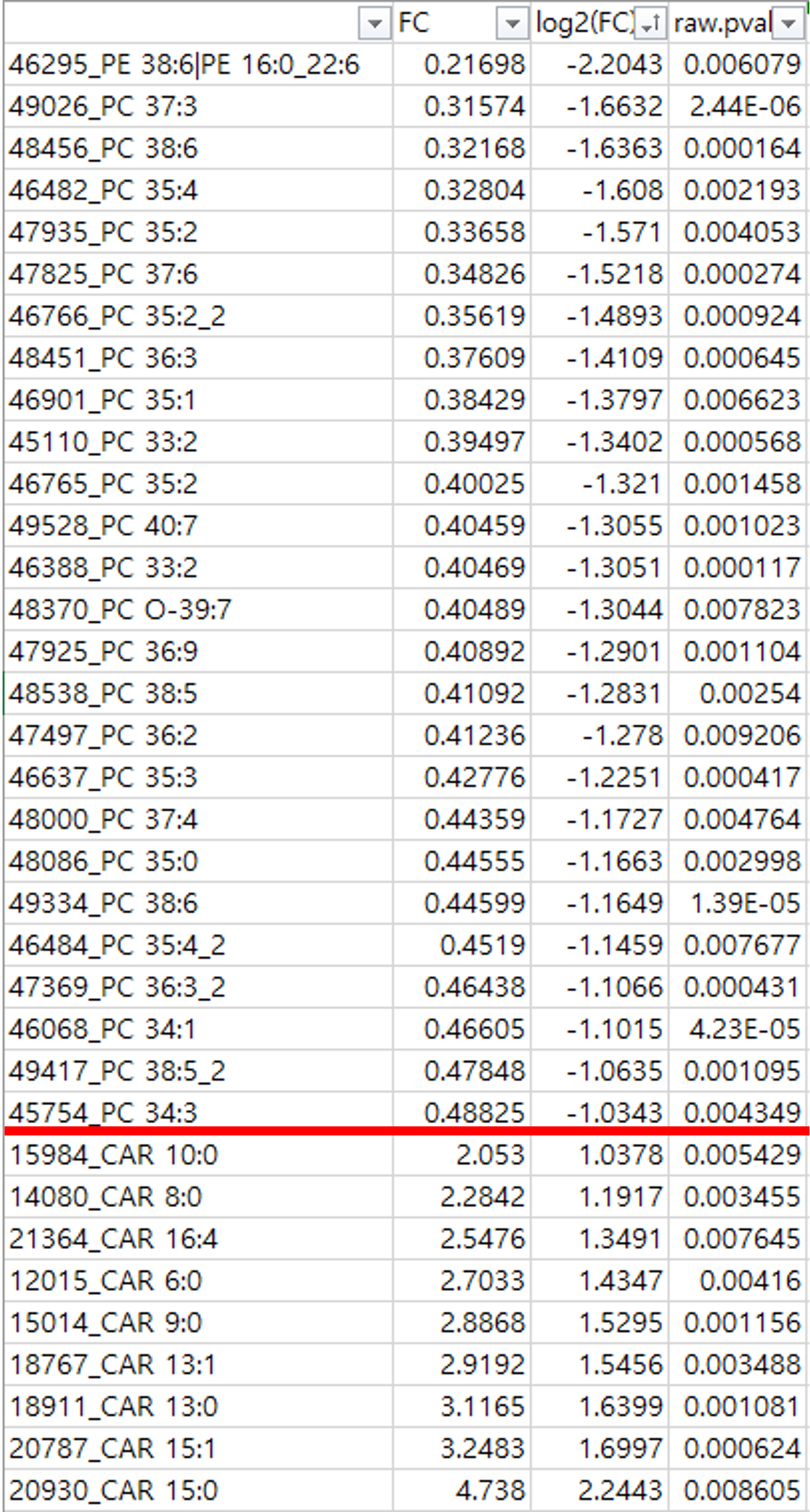

Supplement: S4 Fig — (TIF) [file pone.0345136.s005.tif]

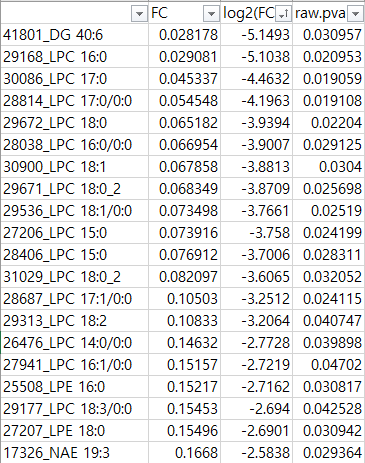

Supplement: S5 Fig — (TIF) [file pone.0345136.s006.tif]
