# Supplementary material for: Lipidomic analysis of bile from patients with extrahepatic cholangiocarcinoma
Source: PLoS One. 2026 Mar 19;21(3):e0345136. doi: 10.1371/journal.pone.0345136 (PMC13001927; doi:10.1371/journal.pone.0345136)
Supplement: Attachment — Submitted filename: Response to Reviewers.docx [file pone.0345136.s008.docx]

**Reviewer #1:**

**1. The most serious concern regarding this study is whether the bile acid abnormality caused the diseases, or whether it occurred as a result of their onset. Please clarify your opinion on this matter.**

Response:

We appreciate the reviewer’s insightful comment regarding the directionality of the association between bile acid abnormalities and disease onset. Although our study does not allow definitive conclusions about causality due to its observational design, we consider the bile-lipid perturbations we observed to be more than epiphenomena, for three following reasons:

1) Biological plausibility/explanation

(1) Phosphatidylcholine (PC) depletion

- Biliary PC plays a protective role for cholangiocytes by acting as a shield against the detergent effects of bile salts and lysophosphatidylcholine (LPC) [1]. *Abcb4(Mdr2)-knock-out* mice, which cannot secrete PC into bile, develop reactive-oxygen-species (ROS) accumulation, chronic cholangitis, and eventually cholangiocarcinoma(CCA) [2,3]. Our finding that PC species were depleted both in extrahepatic CCA(eCCA) and in choledocholithiasis therefore fits a mechanistic model in which early PC loss promotes epithelial injury, chronic inflammation and finally tumorigenesis or lithogenesis, rather than merely reflecting late-stage obstruction.

(2) Diacylglycerol (DG) accumulation.

- DG is a lipid second messenger for PKC, RasGRP and Wnt/β-catenin cascades that drive proliferation and invasion in many cancers. Multi-omics studies demonstrate that diacylglycerol-kinase-α (DGKA) is over-expressed in intrahepatic CCA and that pharmacologic DGKA blockade suppresses tumour growth [4]. This strongly suggests that disordered DG signaling is upstream of, and indispensable for, CCA progression.

(3) Acylcarnitine (CAR) and LPC surges in choledocholithiasis.

- Elevated CAR reflects mitochondrial β-oxidation stress, while LPC initiates NF-κB/IL-6-driven inflammation in cholangiocytes [5]. CAR species have recently been shown to enhance biliary-tract cancer cell motility via JNK activation [6]. Such pro-inflammatory and pro-lithogenic actions are consistent with a causal, not reactive, role.

2) Consistency with previous study

- Consistently, cross-sectional human studies show lower PC concentrations in patients with CCA [7,8].

3) Dose–response alignment within our dataset.

- Within our dataset, both disease groups showed consistent PC depletion versus controls, whereas only the eCCA group displayed a significant rise in selected DG species. Although several PC molecules trended slightly lower in eCCA than in choledocholithiasis, these differences did not reach significance, and DG elevation was largely restricted to eCCA with the exception of DG 40:6. Consequently, the data point to a shared depletion of biliary PC and a disease-specific enrichment of DG in eCCA, rather than a strict step-wise gradient across all three groups. Importantly, these lipid abnormalities were dissociated from bilirubin and ALP, which argues that they cannot be explained solely by the degree of obstruction.

We have inserted a new mechanistic paragraph (lines 305-312), added a concise Discussion section on CAR/LPC biology (lines 337–342), and updated the Conclusions (line 389-393) and references. These changes clarify the rationale while acknowledging residual uncertainty.

**2. In patients with choledocholithiasis and extrahepatic cholangiocarcinoma, bile was collected two to three days after the drainage tube was placed. However, blood test results suggest that, due to drainage, blood test results have not yet normalized at this time, particularly in patients with biliary tract cancer. Please provide evidence to determine the optimal timing for bile collection.**

We thank the reviewer for raising this important point regarding the timing of bile collection after biliary drainage. In our study, bile was collected 2–3 days post-drainage, which represents a clinically feasible window after stabilization of biliary flow, while still minimizing the risk of infection and contamination.

We chose the timing as 2-3 days post-drainage due to following reasons:

1) Clinical stabilization precedes biochemical normalization

- In our cohort, fever, right-upper-quadrant pain, and other cholangitis signs subsided, and oral diet was resumed, by 48–72 h after ENBD. At that same time-point, gross inspection of effluent bile showed clear yellow–green fluid without visible sludge, indicating sufficient wash-out of stagnant bile.

This approach reflects common bedside practice in Korea, where ENBD is generally left in situ for only a few days to minimize tube dislodgement and patient discomfort. A large Chinese series (n = 1016) reported a mean ENBD retention time of 4.0 ± 1.5 days after stone extraction, supporting the practicality of this window [9].

2) Physiologic rationale

The extrahepatic duct holds < 20 mL bile, whereas the liver secretes around 600 mL bile per day. After ductal obstruction is relieved, the bile in ductal column is therefore replaced more than 30 times within the first 24 h. Consequently, local bile chemistry stabilizes well before serum bilirubin normalizes.

3) Previous studies

There are several studies that collected bile using ENBD. However, most of them did not clarify the timing of bile collection [10,11]. One study that specified the timing of bile sampling described it as “24 hours post-procedure.” We extended the interval to 48-72 hr to ensure the clinical resolution of infection, resume oral intake and macroscopically sludge-free bile, before ENBD removal.

Although serum liver tests were still abnormal at sampling, we believe the combination of clinical recovery and visual confirmation of clear bile provides a pragmatic and biologically sound basis for our protocol. We have incorporated this explanation into the *Methods* (line 92-95) and added a limitation sentence in the *Discussion* (line 377-379)

**3. Please provide the following clinicopathological information for patients with extrahepatic cholangiocarcinoma: stage, tumor size, histological type, etc. Please investigate the influence of clinicopathological factors on bile acid composition.**

Response:

Thank you for your insightful comment. We have included a table presenting the individual clinicopathological characteristics of the study cohort (n = 12) (see S1 Table). Given the limited sample size and the heterogeneity of clinical features, we were not able to perform meaningful subgroup analyses or detect statistically significant associations between clinicopathological factors (such as tumor stage, location, or size) and bile acid composition.

Therefore, instead of presenting summary statistics or inferential comparisons, we opted to provide patient-level data to ensure transparency and allow readers to visually assess potential patterns. We agree that future studies with larger and more homogeneous cohorts will be essential to validate any potential associations.

We added the following sentence to Results (line 178-179): Detailed individual clinicopathological data of eCCA patients are provided in S1 Table.

**4. Was hilar bile duct invasion not observed in any of the patients?**

Response:

We included the clinicopathological information for patients with extrahepatic cholangiocarcinoma in S1 Table. Three patients with hilar bile duct cancer was included.

**5. Gallstones are composed of a mixture of cholesterol, calcium salts of bilirubinate or palmitate, proteins, and mucin. Based on their predominant constituents, gallstones can be broadly classified as cholesterol stones, black pigment stones or brown pigment stones. Please Describe the characteristics of stones in patients with choledocholithiasis and explain how these affect bile composition.**

Response:

Thank you for your valuable comment. To address this point, we conducted a detailed review of endoscopic images to assess the characteristics of stones in patients with choledocholithiasis. Based on morphological appearance and color observed during endoscopic retrieval, we categorized the stones into the following subtypes: brown pigment stones (n = 5), black pigment stones (n = 2), mixed pigment stones exhibiting features of both brown and black stones (n = 3), and cholesterol stones (n = 2).

While we attempted to evaluate potential associations between these stone types and the bile acid composition, the sample size in each subgroup was too limited to allow for statistically meaningful comparisons. Furthermore, the heterogeneous nature of the stone composition, including several cases with mixed characteristics, posed additional challenges in stratifying the analysis.

We recognize that stone composition can influence the physicochemical properties of bile, and we fully agree that further investigation with a larger and more homogeneous cohort would be essential to clarify these relationships.

We added the outcomes in *Results* (line 182-187).

**Reviewer #2: The manuscript titled “Lipidomic analysis of bile from patients with extrahepatic cholangiocarcinoma” presents a well-executed investigation into the bile lipidomics of patients with extrahepatic cholangiocarcinoma (eCCA) and choledocholithiasis. The authors demonstrate that distinct lipidomic signatures are associated with these diseases. Although the findings offer valuable insights into the lipid profiles of these conditions, further validation is necessary to draw definitive conclusions. Nevertheless, these results are noteworthy and may contribute to future biomarker discovery efforts.
The following comments are intended to help improve the manuscript:**

**1. Control Group Concerns: The study includes six control subjects with bile duct injury, raising concerns about the representativeness of a truly healthy control group. Additionally, the control group is significantly younger and has lower bilirubin and ALP levels compared to the patient groups. The authors should discuss the rationale behind their choice of controls and whether they consider this group ideal. It is understandable that acquiring samples from completely healthy individuals may be challenging, and if that is the case, the authors should clearly note this.**

We thank the reviewer for this valuable comment regarding the composition of the control group.

As noted, obtaining bile samples from completely healthy individuals is not feasible due to ethical and practical limitations, as bile collection requires invasive procedures such as ERCP or percutaneous drainage. Therefore, we selected patients who required biliary drainage after hepatobiliary surgery as controls, as they had undergone biliary drainage for clinical reasons unrelated to malignancy or chronic biliary disease.

We acknowledge that this group is not fully representative of a "healthy" control population, and that there are differences in age and liver biochemistry (e.g., bilirubin and ALP levels) between the groups. These differences may potentially introduce confounding effects, and we have now explicitly discussed this limitation in the *Discussion* section (line 379-383).

Despite these limitations, the control samples still provide a relevant comparator to evaluate disease-associated bile alterations, particularly in the absence of cancer or chronic inflammation.

**Furthermore, the manuscript should consider whether statistical adjustments could be made to account for differences in age and biochemical parameters, and discuss how these factors may influence the lipidomic profiles.**

We appreciate the reviewer’s thoughtful suggestion regarding statistical adjustment for differences in age and biochemical parameters such as bilirubin and ALP. Due to the relatively small sample size, especially in the control group, we were limited in our ability to perform multivariable regression or covariate-adjusted analyses without risking overfitting. However, we agree that these factors—particularly age and liver function parameters—may influence bile composition and lipidomic profiles.

We have added a statement in the Discussion section acknowledging this limitation and emphasizing the importance of controlling for these potential confounders in future studies with larger cohorts (line 375-377). Despite this limitation, the observed differences in lipidomic signatures remain biologically plausible and are consistent with known pathophysiologic changes in biliary disease.

**2. Presentation of Clinical Data (Table 1): It is strongly recommended to include median values in addition to the mean, especially given the high standard deviations reported. The use of box plots to depict individual data points across groups would enhance the clarity and robustness of the data presentation.**

We sincerely appreciate the reviewer’s thoughtful suggestion. In response, we have revised Table 1 to include median values in parentheses alongside the mean ± standard deviation for all continuous variables, as the data exhibited substantial variability.

Additionally, to enhance data transparency and facilitate visual interpretation, we have created box plots with individual data points overlaid for each variable (S2 Figure). We believe these revisions substantially improve the clarity and robustness of the clinical data presentation.

**3. Data Availability Statement: The manuscript currently states that the data are “available upon reasonable request,” which does not comply with PLOS ONE’s data-sharing policy. It is strongly advised that the authors deposit the lipidomic dataset into a publicly accessible repository and provide an accession number. Since written informed consent has already been obtained from study participants, the authors should clarify why they are not sharing the dataset. Provided that no personal identifiers are disclosed, data sharing is both feasible and encouraged.**

Response:

We thank the reviewer for this important comment. In accordance with PLOS ONE’s data-sharing policy, we agree to make the lipidomic dataset publicly available. The dataset will be uploaded separately. We confirm that all patient data have been fully de-identified and that written informed consent for participation and data sharing was obtained. We appreciate the reviewer’s guidance and are committed to promoting transparency and reproducibility through appropriate data sharing.

**4. Interpretation of Upregulated Lipids: The interpretation of upregulated lipids across groups needs to be more rigorous. Speculative statements should be reframed as hypotheses, and the authors should propose potential validation experiments. Although the manuscript is generally well-written, the discussion section would benefit from further refinement. In particular, redundant content regarding PC and DG should be condensed for clarity.**

Response:

We sincerely thank the reviewer for this thoughtful and constructive comment. In response, we have revised the Discussion section to address all three concerns raised:

1) Reframing speculative statements as hypotheses:

We agree that several mechanistic interpretations in the original draft may have been presented too conclusively. In the revised manuscript, we have carefully reworded these statements to clearly indicate that they represent hypotheses, not definitive conclusions. (line 305-306, 313-315)

Similarly, speculative links between CAR/LPC elevation and mitochondrial stress or inflammation in choledocholithiasis have also been softened and framed as possible mechanisms (line 337-339).

2) Proposing validation experiments:

In line with the reviewer’s suggestion, we included specific experimental approaches that could validate the observed lipidomic changes. These include:

- Use of human-derived cholangiocyte organoid models to test the functional role of DG and DGKA (line 310-312, 326-328)
- Flux analysis using isotope-labeled lipid precursors to track PC-to-DG conversion

These proposals are now explicitly stated in the Discussion section.

3) Condensing redundant content on PC and DG:

We have condensed and reorganized the content related to PC and DG to avoid redundancy and improve clarity. Specifically:

- PC’s physiologic role and its depletion were merged into one concise paragraph.

- DG accumulation and its oncogenic signaling implications were summarized succinctly and placed directly after the PC section to enhance logical flow.

A substantial revision has been made to this section (line 280-281, 288-294, 296-298, 301-304, 360-361). We hope these revisions address the reviewer’s concerns and strengthen the clarity and scientific rigor of the Discussion section.

**5. Consistency in Terminology: The use of terminology throughout the manuscript should be consistent. All abbreviations should be defined at their first mention and used uniformly thereafter.**

We appreciate the reviewer’s helpful suggestion. To enhance consistency, we have ensured that all abbreviations (e.g., ANOVA, LPC, DG, CAR, CBDS) are clearly defined at their first mention. In the main text, we now consistently use the full term “choledocholithiasis” to avoid overuse of abbreviations. However, due to space constraints in figures and tables, we retained the abbreviation “CBDS” for choledocholithiasis in figure legends and table labels. To ensure clarity, we have included abbreviation definitions in all relevant figure captions and table footnotes.

**6. Figure Legends and Supplementary Material: While the figures are informative, their legends could be made clearer to aid interpretation. The authors might consider providing supplementary figures, such as full PCA loading plots or hierarchical clustering dendrograms, to further support their analyses and conclusions.
Overall, the manuscript is novel and clinically relevant, given the understudied nature of bile lipidomics in eCCA patients. Addressing the above comments will significantly strengthen the quality and impact of the manuscript.**

We thank the reviewer for this valuable suggestion. We have carefully revised the figure legends to improve clarity and enhance interpretability (line 208, 224-225, 238-239, and 264-265). Regarding supplementary material, we fully agree that additional figures such as full PCA loading plots and hierarchical clustering dendrograms would further support our findings. However, due to the limitations in sample size and dimensionality reduction methods, we were unable to generate interpretable or publication-quality PCA loading plots or dendrograms in the current dataset. Nonetheless, we appreciate the importance of comprehensive data visualization and will incorporate additional supplementary analyses in future studies as sample sizes expand.

**Reference**

[1]	Ohigashi T, Kanno K, Sugiyama A, Nguyen PT, Kishikawa N, Otani Y, et al. Protective effect of phosphatidylcholine on lysophosphatidylcholine-induced cellular senescence in cholangiocyte. J Hepatobiliary Pancreat Sci 2019;26:568-77. https://doi.org/10.1002/jhbp.684.

[2]	Fickert P, Fuchsbichler A, Wagner M, Zollner G, Kaser A, Tilg H, et al. Regurgitation of bile acids from leaky bile ducts causes sclerosing cholangitis in Mdr2 (Abcb4) knockout mice. Gastroenterology 2004;127:261-74. https://doi.org/10.1053/j.gastro.2004.04.009.

[3]	Huang P, Wei G, Kirkpatrick JD, Lin Y, Tan L, Matta H, et al. Transposon-based oncogene integration in Abcb4(Mdr2)(-/-) mice recapitulates high susceptibility to cholangiocarcinoma in primary sclerosing cholangitis. J Hepatol 2025;82:84-96. https://doi.org/10.1016/j.jhep.2024.07.016.

[4]	Liu W, Wang H, Zhao Q, Tao C, Qu W, Hou Y, et al. Multiomics analysis reveals metabolic subtypes and identifies diacylglycerol kinase α (DGKA) as a potential therapeutic target for intrahepatic cholangiocarcinoma. Cancer Commun (Lond) 2024;44:226-50. https://doi.org/10.1002/cac2.12513.

[5]	Hobbs S, Reynoso M, Geddis AV, Mitrophanov AY, Matheny RW, Jr. LPS-stimulated NF-κB p65 dynamic response marks the initiation of TNF expression and transition to IL-10 expression in RAW 264.7 macrophages. Physiol Rep 2018;6:e13914. https://doi.org/10.14814/phy2.13914.

[6]	Yang Y, Li H, Liu K, Zou L, Xiang S, Geng Y, et al. Acylcarnitines promote gallbladder cancer metastasis through lncBCL2L11-THOC5-JNK axis. Journal of Translational Medicine 2024;22:299. https://doi.org/10.1186/s12967-024-05091-0.

[7]	Hashim Abdalla MS, Taylor-Robinson SD, Sharif AW, Williams HR, Crossey MM, Badra GA, et al. Differences in phosphatidylcholine and bile acids in bile from Egyptian and UK patients with and without cholangiocarcinoma. HPB (Oxford) 2011;13:385-90. https://doi.org/10.1111/j.1477-2574.2011.00296.x.

[8]	Gauss A, Ehehalt R, Lehmann WD, Erben G, Weiss KH, Schaefer Y, et al. Biliary phosphatidylcholine and lysophosphatidylcholine profiles in sclerosing cholangitis. World J Gastroenterol 2013;19:5454-63. https://doi.org/10.3748/wjg.v19.i33.5454.

[9]	Wu J, Wang G. Retention Time of Endoscopic Nasobiliary Drainage and Symptomatic Choledocholithiasis Recurrence After Endoscopic Retrograde Cholangiopancreatography: A Single-center, Retrospective Study in Fuyang, China. Surgical Laparoscopy Endoscopy & Percutaneous Techniques 2022;32:481-7. https://doi.org/10.1097/sle.0000000000001059.

[10]	Ando M, Ito S, Aoki S, Zhang J, Hirano N, Kashiwagi R, et al. Bile liquid biopsy is a useful modality for molecular diagnosis of cancerous tumors in pancreatic head lesions2024.

[11]	Zhang Z, Liu Z, Chen L, Wang Z, Zhai Y, Qian P, et al. Liquid Biopsy-Based Accurate Diagnosis and Genomic Profiling of Hard-to-Biopsy Tumors via Parallel Single-Cell Genomic Sequencing of Exfoliated Tumor Cells. Anal Chem 2024;96:14669-78. https://doi.org/10.1021/acs.analchem.4c03462.
