# Supplementary material for: Lipidomic analysis of bile from patients with extrahepatic cholangiocarcinoma
Source: PLoS One. 2026 Mar 19;21(3):e0345136. doi: 10.1371/journal.pone.0345136 (PMC13001927; doi:10.1371/journal.pone.0345136)
Supplement: Attachment — Submitted filename: Response_to_Reviewers_auresp_2.docx [file pone.0345136.s009.docx]

**Reviewer #2: The authors have addressed many of the points raised in the previous review of the manuscript “Lipidomic analysis of bile from patients with extrahepatic cholangiocarcinoma”, which is appreciated. However, there are still a few important issues that need to be resolved:
1. Data Availability:
The authors mention that the lipidomic dataset has been deposited in a public repository, which is a positive step. However, the manuscript currently lacks the specific accession number, which is essential for readers to access the data. Additionally, the statement that “all data are located within the manuscript” is inaccurate and should be revised. Please ensure that:
• The manuscript clearly states that the lipidomic dataset is publicly available.
• The name of the repository and the corresponding accession number are included.**

We sincerely thank the reviewer for highlighting this important point. We would like to clarify a possible misunderstanding in our previous response. The statement *“The dataset will be uploaded separately”* was intended to mean that the complete lipidomic dataset is provided as supplementary files to the manuscript, not that it was deposited in a separate public repository. In the previous revised-Data Availability Statement, we have specified that “All relevant data are within the manuscript and its Supporting Information files.” We deeply apologize if our earlier wording caused any confusion.

In accordance with PLOS ONE’s data-sharing policy, all relevant lipidomic data are fully included within the manuscript and its Supporting Information files. The dataset is de-identified, and written informed consent for participation and data sharing was obtained from all participants. We confirm that no external repository deposition was performed, as the complete dataset is already accessible within the supplementary materials.

**2. Use of AI Tools:
It seems that sections of the manuscript may have been revised or edited using AI-based tools such as ChatGPT. If that is the case, the use of such tools should be acknowledged transparently in the Acknowledgements section, just before the References, in line with current publication practices.**

We sincerely appreciate the reviewer’s thoughtful comment. We confirm that ChatGPT was used during the preparation of this manuscript solely for language editing and to improve clarity of expression. In line with the journal’s policy, we will add an explicit statement in the Acknowledgements section.

*The authors declare that OpenAI’s ChatGPT (version GPT-4.5, OpenAI, San Francisco, CA, USA) was used for language editing and improvement of readability. All scientific content, analysis, and interpretations are the sole responsibility of the authors.*

**3. Language and Clarity:
While the language has generally improved, there are still a few grammatical issues that need attention. For example, there appears to be a missing word or phrase around Line 383, which affects the readability of that sentence. A thorough proofread is recommended to ensure the manuscript is clear and free of such minor errors.**

We sincerely thank the reviewer for the careful reading and constructive feedback. We carefully reviewed Line 383 and identified that a missing word affected the readability of the sentence. This has been corrected to ensure clarity. In addition, we performed a thorough proofread of the entire manuscript to address minor grammatical issues and improve overall readability. We believe these revisions have enhanced the clarity and flow of the manuscript.

- Line 43: added “more”
- Line 111: added “for the”
- Line 271: revised from” “…demonstrated significant alterations in lipid concentrations in the bile of patients with pathological conditions of eCCA and choledocholithiasis, than in the bile of controls.” to
  “…demonstrated significant alterations in bile lipid concentrations in patients with eCCA and choledocholithiasis compared with controls.”
- Line 383: from “may have introduced confounding.” to
  “may have introduced potential confounding effects.”

**Reviewer #3: All comments have been addressed. The manuscript meets the qualification of PLOS one. I recommend this manuscript to be published.**

We are sincerely grateful to Reviewer #3 for the positive and encouraging evaluation. We greatly appreciate your recommendation for publication. We thank you for your constructive comments throughout the review process, which have helped us significantly improve the manuscript.
